# The association between care-resistant behavior in dementia patients and nurses’ occupational burnout: a cross-sectional serial mediation analysis of psychological detachment and professional calling

**DOI:** 10.3389/fpsyg.2026.1860688

**Published:** 2026-09-28

**Authors:** Yao Ding, Ying Shen, Aini Lv, Le Lu, Pan Luo, Yan Song

**Affiliations:** Department of Rehabilitation, Tangdu Hospital, the Fourth Military Medical University, Shaanxi, China

**Keywords:** care-resistant behavior, cross-sectional serial mediation, occupational burnout, professional calling, psychological detachment

## Abstract

**Background:**

Care-resistant behavior in dementia patients is a common and highly challenging stressor in clinical nursing practice. It not only increases the difficulty of nursing care but may also have a negative impact on nurses’ occupational health. Occupational burnout, a prevalent state of psychological exhaustion among nurses, poses a serious threat to both the mental health of nursing staff and the quality of nursing services. Psychological detachment, as an important experience of recovery from the work context, and professional calling, as an individual’s intrinsic identification with and sense of meaning in their profession, may play key mediating roles in the relationship between care-resistant behavior and occupational burnout. However, existing research has yet to systematically explore the serial indirect associations among these variables. Therefore, this study used a chain mediation model grounded in the Job Demands-Resources (JD-R) theory to examine the associations among care-resistant behavior in dementia patients, psychological detachment, professional calling, and occupational burnout, including the hypothesized serial indirect pathway, aiming to provide empirical evidence for nursing managers to develop targeted intervention strategies.

**Methods:**

A questionnaire survey was conducted among 421 nurses recruited from five tertiary hospitals located in Shaanxi Province, China. The survey utilized a general information questionnaire, the Chinese version of the Resistiveness to Care Scale for patients with dementia, the Psychological Detachment Questionnaire, Professional Calling Scale (PCS), and the Burnout Inventory. Since all continuous variables did not satisfy the assumption of normal distribution (assessed by the Shapiro–Wilk test), Spearman correlation analysis was used to examine the relationships among variables, and structural equation modeling (SEM) using AMOS with the Bootstrap method (2,000 resamples) was employed to estimate the direct and indirect associations specified in the hypothesized serial mediation model, with the significance of indirect effects evaluated using 95% confidence intervals.

**Results:**

In this study, the mean scores for care-resistant behavior, psychological detachment, professional calling, and occupational burnout among the 421 nurses were (43.89 ± 21.75), (9.61 ± 3.72), (31.03 ± 10.96), and (63.00 ± 27.79), respectively. Care-resistant behavior was positively correlated with occupational burnout (*r* = 0.702, *p* < 0.001), and negatively correlated with psychological detachment (*r* = −0.519, *p* < 0.001) and professional calling (*r* = −0.626, *p* < 0.001). Psychological detachment was positively correlated with professional calling (*r* = 0.600, *p* < 0.001) and negatively correlated with occupational burnout (*r* = −0.641, *p* < 0.001). Professional calling was negatively correlated with occupational burnout (*r* = −0.862, *p* < 0.001). Serial mediation analysis indicated that both the direct association (*β* = 0.303, *p* = 0.001) and the total indirect effect (*β* = 0.510, *p* = 0.001) between care-resistant behavior in dementia patients and nurses’ occupational burnout were significant. A significant serial indirect association involving psychological detachment and professional calling was observed in the relationship between care-resistant behavior and occupational burnout (*β* = 0.126). This serial indirect association accounted for 15.5% of the total association, with a 95% confidence interval of (0.075–0.201).

**Conclusion:**

This study identified a statistically significant serial indirect association involving psychological detachment and professional calling in the relationship between care-resistant behavior in dementia patients and occupational burnout among clinical nurses. The observed pattern was consistent with the hypothesized serial mediation model but did not establish the temporal or causal ordering of these variables. Accordingly, the findings should be interpreted as associations rather than causal relationships. The results provide a theoretical basis for future longitudinal and intervention studies examining whether improving psychological detachment and professional calling may be associated with lower occupational burnout.

## 1 Introduction

Dementia, often referred to as major neurocognitive disorder, is a clinical syndrome caused by various underlying etiologies. Its core feature is an acquired, persistent decline in multiple higher cognitive functions due to brain disease, with a severity sufficient to impair an individual’s independence in daily living and social or occupational functioning (Loi et al., 2023). As the global population continues to age, the number of people living with dementia is projected to increase substantially, from 57.4 million in 2019 to 152.8 million in 2050 (GBD 2019 Dementia Forecasting Collaborators, 2022). The number of dementia patients in China accounts for approximately one-quarter of the global total, ranking first in the world. It is projected that by 2050, the number of dementia cases worldwide will increase to 152 million (Alzheimer’s Association, 2023), posing a severe challenge to healthcare systems, particularly long-term care facilities (Lee et al., 2024). In these facilities, nurses serve as the core workforce providing professional care. However, due to progressive cognitive decline, individuals with dementia often experience neuropsychiatric symptoms. Among these, care-resistant behavior (CRB)—including verbal or physical resistance such as refusing to eat, take medication, or bathe, as well as verbal or physical aggression (Bratches et al., 2025)—has become one of the most common and challenging issues in the care process (Xiao et al., 2024). Such behaviors not only severely impede the smooth implementation of care plans and affect patients’ quality of life and safety, but also constitute a significant source of work stress for the clinical nurses who directly provide care (El Gedaily et al., 2024; Xiao et al., 2021). Nurses directly and frequently encounter these behaviors. Prolonged exposure to such a high-stress work environment not only imposes substantial physical exhaustion but also involves persistent psychological experiences of frustration, helplessness, and emotional impact. This makes them highly susceptible to emotional exhaustion, placing them at significant risk for occupational burnout (Adriaenssens et al., 2015). Therefore, care-resistant behavior in dementia patients is regarded as a potential correlate of occupational burnout among clinical nurses. Occupational burnout is a psychological syndrome triggered by prolonged work-related stress, characterized by emotional exhaustion, depersonalization, and reduced personal accomplishment (Zhang et al., 2023). It has become a prominent public health issue affecting the physical and mental well-being of nursing staff as well as their career stability (Sohrabi et al., 2022). Multiple studies have shown that the detection rate of occupational burnout among nurses both domestically and internationally generally exceeds 30%, and in some intensive care units, psychiatric departments, and geriatric nursing populations, it is even as high as over 50% (Friganović et al., 2019; Quesada-Puga et al., 2024). Persistent occupational burnout not only impairs nurses’ job satisfaction and professional efficacy, but may also affect the quality of nursing care and patient safety (Salvagioni et al., 2017), and even trigger high turnover intention among nursing staff, further exacerbating the structural shortage of nursing human resources (Makara-Studzińska et al., 2019). Therefore, an in-depth exploration of the relationships linking care-resistant behavior in dementia patients with nurses’ occupational burnout holds significant theoretical and practical importance for maintaining nurses’ mental health and improving the quality of care.

The Job Demands-Resources Model (JD-R) provides an integrative framework for explaining this process. According to this theory, any occupational context comprises two types of characteristics: job demands and job resources (Demerouti et al., 2001). Job demands refer to work factors that require sustained physical and psychological effort and are associated with certain physiological and psychological costs. Job resources, on the other hand, help achieve work goals, reduce job demands, or promote personal growth and development (Bakker and Demerouti, 2017). The model proposes that high job demands lead to stress and health problems (the health impairment process), while adequate resources can motivate employees to enhance work motivation and productivity (the motivational process). According to this theory, when job demands are high and job resources are insufficient, individuals are prone to emotional exhaustion, thereby leading to burnout (Crawford et al., 2010). However, empirical research has not examined the mechanism by which stress-induced loss of job resources contributes to the development of occupational burnout among clinical nurses.

The path from demands to burnout is not a simple linear relationship; rather, it involves complex psychological processes. The JD-R model also points out that job resources do not merely function as direct buffering variables, but are more likely to mediate the relationship between job demands and occupational burnout (Stan and Ciobanu, 2022). That is, when facing high job demands, whether individuals can effectively utilize or activate internal and external resources directly influences their stress perception and level of burnout. In high-pressure work settings within the nursing field, psychological detachment and professional calling serve as two key psychological resources that help individuals cope with challenges and achieve goals. Psychological detachment refers to the active disengagement from work-related cognitive and emotional connections during non-work hours, ceasing to think about or worry about work matters, thereby facilitating the recovery of physiological and psychological resources (Karabinski et al., 2021). Clinical nurses face high-intensity work pressure, life-and-death decisions, complex interpersonal relationships, and substantial emotional investment on a daily basis. This sustained physical and emotional involvement, coupled with emotional consumption, makes it easy for work stress to encroach upon their personal lives, leading to occupational burnout, anxiety, depression, and physical and mental health issues (Wu et al., 2023). Psychological detachment is a key element in interrupting this vicious cycle. Research has shown that psychological detachment promotes the restoration of individuals’ cognitive and emotional resources, helps maintain their physical and mental health and ensures patient safety, and is crucial for maintaining work-life balance and reshaping professional identity and sense of meaning, thereby alleviating occupational burnout (Wu et al., 2023; Zhang et al., 2023).

Professional calling refers to an individual’s strong passion for their chosen profession, along with a profound sense of meaning and responsibility derived from their work (Zhang, 2024). This elevated sense of meaning can translate into powerful psychological motivation to cope with challenges, buffering the negative effects of external stress and thereby reducing emotional exhaustion (Liu et al., 2024). Research indicates that a strong professional calling is generally regarded as an important psychological resource for resisting occupational burnout. Individuals with a high sense of professional calling tend to demonstrate greater work engagement, job satisfaction, and well-being, while exhibiting relatively lower levels of occupational burnout (Wang and Yuan, 2025). Therefore, as a stable personal resource, professional calling is hypothesized to be negatively associated with occupational burnout.

In summary, although care-resistant behavior in dementia patients, psychological detachment, and professional calling have each been associated with occupational burnout, the relationships among these factors have not been sufficiently explored. To construct a framework for understanding how care-resistant behavior in dementia patients may be linked to nurses’ occupational burnout, we drew upon the JD-R theory. Within this framework, care-resistant behavior in dementia patients, as a persistent emotional and physical demand, may be associated with greater difficulty recovering after work and lower levels of psychological detachment (Muhamad Nasharudin et al., 2020). Lower psychological detachment may, in turn, be associated with weaker professional calling, a core personal resource for nurses (Deng and Chen, 2019). Weaker professional calling may be associated with higher levels of occupational burnout (Liu et al., 2025). Based on this integrated theoretical framework, we hypothesize that psychological detachment and professional calling may serve as chain mediators in the relationship between care-resistant behavior in dementia patients and occupational burnout. The hypothesized ordering of these variables was theoretically specified on the basis of the Job Demands-Resources (JD-R) model rather than inferred from temporal evidence in the present data. Specifically, care-resistant behavior was conceptualized as a persistent job demand that may consume nurses’ psychological and emotional resources; psychological detachment was conceptualized as a recovery-related experience reflecting the ability to disengage mentally from work-related demands; and professional calling was viewed as a meaning-related personal resource reflecting occupational purpose and motivation. From this perspective, greater exposure to care-resistant behavior may be associated with poorer psychological detachment, which may in turn be associated with weaker professional calling and higher occupational burnout. Importantly, given the cross-sectional design, this proposed sequence should be understood as a theory-guided framework for examining contemporaneous associations rather than as an empirically established temporal or causal process.

This study proposes two hypotheses (Figure 1):

**Figure 1:**
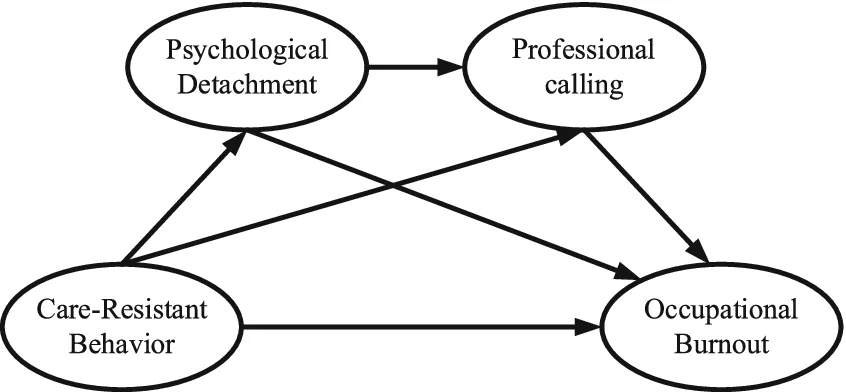
Conceptual model.

*H1*: Care-resistant behavior in dementia patients is positively associated with nurses' occupational burnout.

*H2*: Psychological detachment and professional calling form a hypothesized serial indirect pathway in the association between care-resistant behavior in dementia patients and nurses' occupational burnout.

## 2 Methods

### 2.1 Study design and participants

In this cross-sectional study, a convenience sampling method was used to recruit eligible nurses from five tertiary hospitals located in Shaanxi Province, China, between July and December 2025. The participating hospitals were all tertiary-level medical institutions, including tertiary first-class hospitals, and were located in different regions of Shaanxi Province. The inclusion criteria were: (1) registered clinical nurses holding a valid nurse practice certificate; (2) having at least 3 months of nursing experience with dementia patients (including departments such as geriatrics, psychiatry, rehabilitation, neurology, and nursing homes); (3) having encountered care-resistant behavior in patients with dementia at least twice per week during the preceding 3 months, thereby ensuring sufficient recent experience to evaluate such behavior; (4) voluntary participation in this study with a commitment to complete the questionnaires and cooperate with data collection throughout the entire process. The exclusion criteria were: (1) nurses primarily engaged in administrative roles, such as nursing administrators or head nurses; (2) those who had experienced major life events (e.g., death of a relative, major illness) within the past 6 months, or those with existing mental or psychological disorders; (3) those concurrently participating in other clinical trials involving severe psychological stress or interventions targeting occupational burnout. This operational threshold was adopted to ensure that participants could provide experience-based ratings of care-resistant behavior; it was not intended to estimate the prevalence of care-resistant behavior exposure among the broader nursing population. Therefore, this inclusion criterion resulted in an exposure-enriched sample, in which nurses with more frequent exposure to care-resistant behavior were preferentially included.

The sample size for this study was calculated following the guidelines proposed by Kline (Fonseca, 2013). These guidelines recommend that the sample size should be 10 to 20 times the number of study variables. Since this study involved 23 variables, and considering a 15% rate of invalid questionnaires, the required sample size was estimated to be between 271 and 542 participants. This study ultimately surveyed 438 participants.

### 2.2 Research instruments

#### 2.2.1 General information questionnaire

The questionnaire was self-designed by the researchers after reviewing the literature, including: gender, age, years of work experience, marital status, education level, professional title, employment type, average monthly income, frequency of night shifts, hospital level, and work department.

#### 2.2.2 Chinese version of the resistiveness to care scale for patients with dementia, RTC-C

In this study, the Chinese version of the Care-Resistant Behavior in Dementia Patients Scale, translated and revised by scholar Wang (2024), was used. This scale consists of four dimensions with a total of 13 items. The score ranges from 0 to 156 points. Generally, a higher score indicates more severe care-resistant behaviors encountered by the respondent. This scale has good structural validity, and the Cronbach’s alpha coefficient is 0.867. In this study, the Cronbach’s alpha coefficient for this scale was 0.912.

#### 2.2.3 Psychological detachment questionnaire, PDQ

In this study, the Psychological Detachment subscale from the Recovery Experience Questionnaire developed by Sonnentag and Fritz (2007) was used. This subscale has a unidimensional structure consisting of four items, rated on a 5-point Likert scale ranging from “strongly disagree” to “strongly agree,” with scores assigned from 1 to 5 points. The total score ranges from 4 to 20 points, with higher scores indicating a higher level of psychological detachment. The Cronbach’s α coefficient for the scale was 0.84, and the KMO value was 0.74, indicating that the scale has good reliability and validity. In this study, the Cronbach’s alpha coefficient for this scale was 0.811.

#### 2.2.4 Professional calling scale, PCS

In this study, the Professional Calling Scale developed by scholar Zhang (2015) in 2015 was used. This scale consists of three dimensions: altruistic contribution, directive force, and meaning and value, comprising a total of 11 items. The scale adopts a 5-point Likert scoring system, ranging from “completely inconsistent” to “completely consistent,” with scores assigned from 1 to 5 points. The total score ranges from 11 to 55 points, with higher scores indicating a stronger professional calling. The Cronbach’s α coefficient for this scale was 0.84, demonstrating good reliability and validity. In this study, the Cronbach’s alpha coefficient for this scale was 0.933.

#### 2.2.5 Burnout inventory

This study employed the Chinese version of the Occupational Burnout Inventory, as revised by the scholar Li et al. (2003). This scale consists of three dimensions: emotional exhaustion, depersonalization, and reduced personal accomplishment, comprising a total of 22 items. A 7-point Likert scale is adopted, with scores ranging from 0 (“never”) to 6 (“every day”). The total score ranges from 0 to 132 points, with higher scores indicating more severe occupational burnout. The Cronbach’s α coefficient for this scale was 0.738, demonstrating good reliability and validity. In this study, the Cronbach’s alpha coefficient for this scale was 0.949.

### 2.3 Data collection

In this study, data collection was conducted after obtaining approval from the Ethics Committee of The Second Affiliated Hospital of Air Force Military Medical University. The researchers uploaded the selected scales to the Questionnaire Star and generated corresponding questionnaire links. Prior to the initiation of the study, the research team reached out to the nursing administrators or head nurses from the relevant departments across the five participating hospitals. A comprehensive explanation was given regarding the objectives, significance, and proper completion procedures of the survey, and assurances of anonymity and data protection were explicitly provided. With the assistance of these coordinators, the questionnaire link was disseminated through departmental WeChat groups to all eligible nursing staff. After obtaining their understanding and support, the head nurses were responsible for sharing the Questionnaire Star links to the departmental WeChat groups for eligible participants to complete. To ensure the quality of the questionnaire data and avoid omissions or random responses, all items were set as mandatory, and the same IP address was restricted to completing the questionnaire only once via WeChat. All survey data were automatically collected through the WJX system and exported in Excel format. A total of 438 questionnaires were collected. After rigorous review by two individuals to exclude unqualified and invalid questionnaires, 421 valid questionnaires remained. The proportion of valid questionnaires was 96.1% (421/438), which represents the valid-questionnaire rate rather than the true response rate because the number of nurses who received the survey invitation but did not complete the questionnaire was not prospectively recorded.

### 2.4 Statistical analyses

In this study, IBM SPSS 27.0 and AMOS 24.0 software were used for data analysis and construction of the mediation model. Categorical data were expressed as frequencies and percentages, while continuous data were expressed as mean ± standard deviation. The normality of continuous variables was assessed before statistical analysis using the Shapiro–Wilk test. The degree of non-normality was further evaluated by examining skewness and kurtosis values. Although all continuous variables showed significant deviations from normality according to the Shapiro–Wilk test (*p* < 0.05), the skewness values ranged from −0.419 to 0.715 and kurtosis values ranged from −1.359 to 2.096, indicating mild-to-moderate deviations from normality rather than severe departures (Supplementary Table S1). Since all continuous variables did not satisfy the assumption of normal distribution (*p* < 0.05), non-parametric statistical methods were adopted. Comparisons between two groups were performed using the Mann–Whitney U test, whereas comparisons among three or more groups were conducted using the Kruskal–Wallis test. Spearman correlation analysis was used to examine the relationships among variables, with *p* < 0.05 indicating statistically significant correlations. Multiple linear regression analysis was conducted to examine the associations of care-resistant behavior in dementia patients, psychological detachment, and professional calling with nurses’ occupational burnout levels. This analysis was performed as a supplementary analysis to describe the independent associations among the main study variables and was not used for variable selection or determination of the SEM structure. The hypothesized serial mediation model was specified *a priori* based on the Job Demands-Resources theory rather than derived from regression results. AMOS 24.0 was used to construct the hypothesized serial mediation model. The SEM framework treated care-resistant behavior, psychological detachment, professional calling, and occupational burnout as latent constructs represented by their corresponding observed indicators rather than simple scale scores. Although non-parametric methods were applied in preliminary analyses due to deviations from normality, maximum likelihood (ML) estimation was retained for SEM because previous methodological studies have demonstrated that ML estimation can provide reliable parameter estimates when the sample size is adequate and the degree of non-normality is mild to moderate. In addition, the bootstrap method was applied to estimate confidence intervals for indirect effects, which reduces the reliance on normality assumptions of indirect-effect distributions. The Bootstrap method (with 2,000 resamples) was employed to evaluate the mediation effects (95% CI, *p* < 0.05) for validation. The *p*-value indicated the level of statistical significance, with a *p*-value less than 0.05 considered statistically significant. The three specific indirect effects were calculated as a_1_b_1_, a_2_b_2_, and a_1_d_21_b_2_, respectively. The total indirect effect was calculated as a_1_b_1_ + a_2_b_2_ + a_1_d_21_b_2_, and the total effect was calculated as the sum of the direct effect and the total indirect effect. The proportion attributable to each effect was calculated using the standardized estimate according to the formula: effect/total effect × 100%. For additional transparency, the proportion of each specific indirect effect relative to the total indirect effect was also calculated. An indirect effect was considered statistically significant when its 95% bootstrap confidence interval did not include zero. All effect decompositions and percentage calculations were performed using standardized estimates to ensure that the direct and indirect effects were expressed on the same metric. Accordingly, Table 1 presents standardized effect estimates, bootstrap standard errors, and 95% bootstrap confidence intervals.

**Table 1:** Mediation effect analysis (Standardized).

Path	*β*	Boot SE	Bootstrap 95% CI	Proportion of the total effect (%)	Percentage of total indirect effect (%)
Total effect	0.813	0.037	0.734 ~ 0.881	100	—
Direct effect (Care-Resistant Behavior → Occupational Burnout)	0.303	0.062	0.185 ~ 0.418	37.3	—
Indirect effect	0.510	0.057	0.397 ~ 0.624	62.7	100
Care-Resistant Behavior → Psychological Detachment → Occupational Burnout	0.044	0.024	−0.001 ~ 0.090	5.4	8.6
Care-Resistant Behavior → Professional Calling → Occupational Burnout	0.340	0.042	0.264 ~ 0.434	41.8	66.7
Care-Resistant Behavior → Psychological Detachment → Professional Calling → Occupational Burnout	0.126	0.031	0.075 ~ 0.201	15.5	24.7

The total indirect effect was calculated as 0.044 + 0.340 + 0.126 = 0.5100.044 + 0.340 + 0.126 = 0.5100.044 + 0.340 + 0.126 = 0.510. The total effect was calculated as 0.303 + 0.510 = 0.8130.303 + 0.510 = 0.8130.303 + 0.510 = 0.813. The percentage of the total effect was calculated as standardized effect/0.813 × 100%, whereas the percentage of the total indirect effect was calculated as specific indirect effect/0.510 × 100%. Standardized estimates were used consistently to avoid combining coefficients expressed on different measurement scales.

## 3 Results

### 3.1 General demographic data analysis

The general demographic data analysis of participants is presented in Table 2. Among the 421 nurses, the majority were female (86.9%) and aged between 32 and 41 years (43.9%). More than half of the participants had 6 to 15 years of work experience (52.5%), and 70.1% were married. In terms of educational level, the majority held a bachelor’s degree (73.6%), while the proportions of those with associate degrees (11.4%) and master’s degrees or above (15%) were relatively lower. Nearly half of the nurses held the title of primary nurse (42.3%), while those with deputy senior titles or above accounted for a relatively small proportion (5%). Regarding employment type, the majority were contract-based employees (58%), while those with permanent positions accounted for the smallest proportion (11.4%). The most common average monthly income was between 5,000 and 8,000 RMB (39.7%), and a monthly night shift frequency of 6 to 10 shifts was the most common (38.2%). The most frequently represented department among participants was rehabilitation medicine (38.7%), while the proportion of participants from other departments was notably limited, representing only 1.2% of the total sample.

**Table 2:** General demographic information of nurses (*n* = 421).

Variables	Items	*n*	Constituent ratio (%)	*p*
Gender	Male	55	13.1	0.046^a^
	Female	366	86.9	
Age	20 ~ 31	158	37.5	<0.001^b^
	32 ~ 41	185	43.9	
	42 ~ 51	62	14.7	
	>51	16	3.8	
Years of work experience	≤5	138	32.8	<0.001^b^
	6 ~ 15	221	52.5	
	≥16	62	14.7	
Marital status	Unmarried	124	29.5	<0.001^b^
	Married	295	70.1	
	Other	2	0.5	
Education level	Associate Degree	48	11.4	<0.001^b^
	Bachelor’s Degree	310	73.6	
	Master’s Degree or Above	63	15	
Professional title	Nurse	83	19.7	<0.001^b^
	Primary Nurse	178	42.3	
	Supervisor Nurse	139	33	
	Associate Chief Nurse or Above	21	5	
Employment type	Permanent staff	48	11.4	<0.001^b^
	Contract-based employee	244	58	
	Human resources agency employee	85	20.2	
	Dispatch employee	44	10.5	
Average monthly income	<5,000	32	7.6	<0.001^b^
	5,000 ~ 8,000	167	39.7	
	8,000 ~ 11,000	163	38.7	
	>11,000	59	14	
Frequency of night shifts	0	57	13.5	<0.001^b^
	1 ~ 5	150	35.6	
Variables	Items	n	Constituent ratio (%)	*P*
	6 ~ 10	161	38.2	
	>10	53	12.6	
Department	Neurology department	82	19.5	<0.001^b^
	Neurosurgery department	49	11.6	
	Geriatrics department	66	15.7	
	Psychiatry department	55	13.1	
	Rehabilitation department	163	38.7	
	General medicine department	1	0.2	
	Other	5	1.2	

a Mann–Whitney U.

b Kruskal–Wallis Test.

In this study, a non-parametric test was conducted to compare the general demographic data with the total occupational burnout scores. The results showed that differences in occupational burnout scores were statistically significant (*p* < 0.05) for gender, age, years of work experience, marital status, educational level, professional title, employment type, average monthly income, night shift frequency, and work department. However, the difference in occupational burnout scores across different hospital levels was not statistically significant (*p* > 0.05).

### 3.2 Common method Bias test

The Harman single-factor test was used to conduct factor analysis on the items involved in this study. The results showed that there were a total of 10 factors with eigenvalues greater than 1, and the variance explained by the first factor was 36.95%, which is below the critical threshold of 40% (Podsakoff et al., 2012), suggesting that no dominant single-factor structure was evident. However, given the recognized limitations of the Harman single-factor test, this result should not be interpreted as definitive evidence that common method bias is absent. Therefore, common method bias was further considered through the measurement model of SEM and addressed in the interpretation of the findings.

### 3.3 Correlation analysis of care-resistant behavior in dementia patients, psychological detachment, professional calling, and occupational burnout

The results of the correlation analysis showed that care-resistant behavior was positively correlated with occupational burnout (*r* = 0.702, *p* < 0.001), while negatively correlated with psychological detachment (*r* = −0.519, *p* < 0.001) and professional calling (*r* = −0.626, *p* < 0.001). These findings indicate that nurses who experience more severe care-resistant behaviors from dementia patients tend to report higher levels of occupational burnout. See Table 3.

**Table 3:** Correlations among care-resistant behavior, psychological detachment, professional calling, and occupational burnout (*n* = 421).

Item	Care-resistant behavior	Psychological detachment	Professional calling	Occupational burnout
Care-resistant behavior	1.000	—	—	—
Psychological detachment	−0.519**	1.000	—	—
Professional calling	−0.626**	0.600**	1.000	—
Occupational burnout	0.702**	−0.641**	−0.862**	1.000

**P < 0.001.

### 3.4 Multiple linear regression analysis of factors associated with nurses’ occupational burnout

In this study, multiple linear regression analysis was conducted to examine the associations of care-resistant behavior in dementia patients, psychological detachment, and professional calling with nurses’ occupational burnout (see Table 4). The results showed that care-resistant behavior was positively associated with nurses’ occupational burnout (*p* < 0.001), while psychological detachment and professional calling were negatively associated with nurses’ occupational burnout (*p* < 0.001). Together, these three variables explained 79.8% of the variance in occupational burnout. Additionally, the variance inflation factor (VIF) values were all less than 5, indicating no multicollinearity among the variables.

**Table 4:** Multiple linear regression analysis of nurses’ occupational burnout levels.

Variable	B	*β*	*t*	R*^2^*	*p*	VIF
Level 1 (Constant)	23.642		10.855	49.3	<0.001	
Care-resistant behavior	0.897	0.702	20.165		<0.001	1.000
Level 2 (Constant)	61.809		14.917	59.7	<0.001	
Care-resistant behavior	0.646	0.505	13.924		<0.001	1.368
Psychological detachment	−2.826	−0.379	−10.428		<0.001	1.368
Level 3 (Constant)	109.630		29.110	79.8	<0.001	
Care-resistant behavior	0.299	0.234	8.053		<0.001	1.736
Psychological detachment	−1.053	−0.141	−4.988		<0.001	1.649
Professional calling	−1.599	−0.630	−20.322		<0.001	1.984

### 3.5 Direct and indirect associations involving psychological detachment and professional calling in the relationship between care-resistant behavior in dementia patients and nurses’ occupational burnout

In this study, AMOS 24.0 was used to construct a chain mediation model, with care-resistant behavior in dementia patients as the independent variable, psychological detachment and professional calling as mediating variables, and nurses’ occupational burnout level as the dependent variable. The maximum likelihood method was used to test the model parameters. The results showed: CMIN/DF = 2.466, GFI = 0.931, RMR = 0.038, AGFI = 0.912, RMSEA = 0.059, CFI = 0.948, TLI = 0.936, and the 90% confidence interval of RMSEA was 0.051–0.067, indicating an acceptable model fit. The constructs in the SEM were specified as latent variables represented by their corresponding observed indicators. See Figure 2.

**Figure 2:**
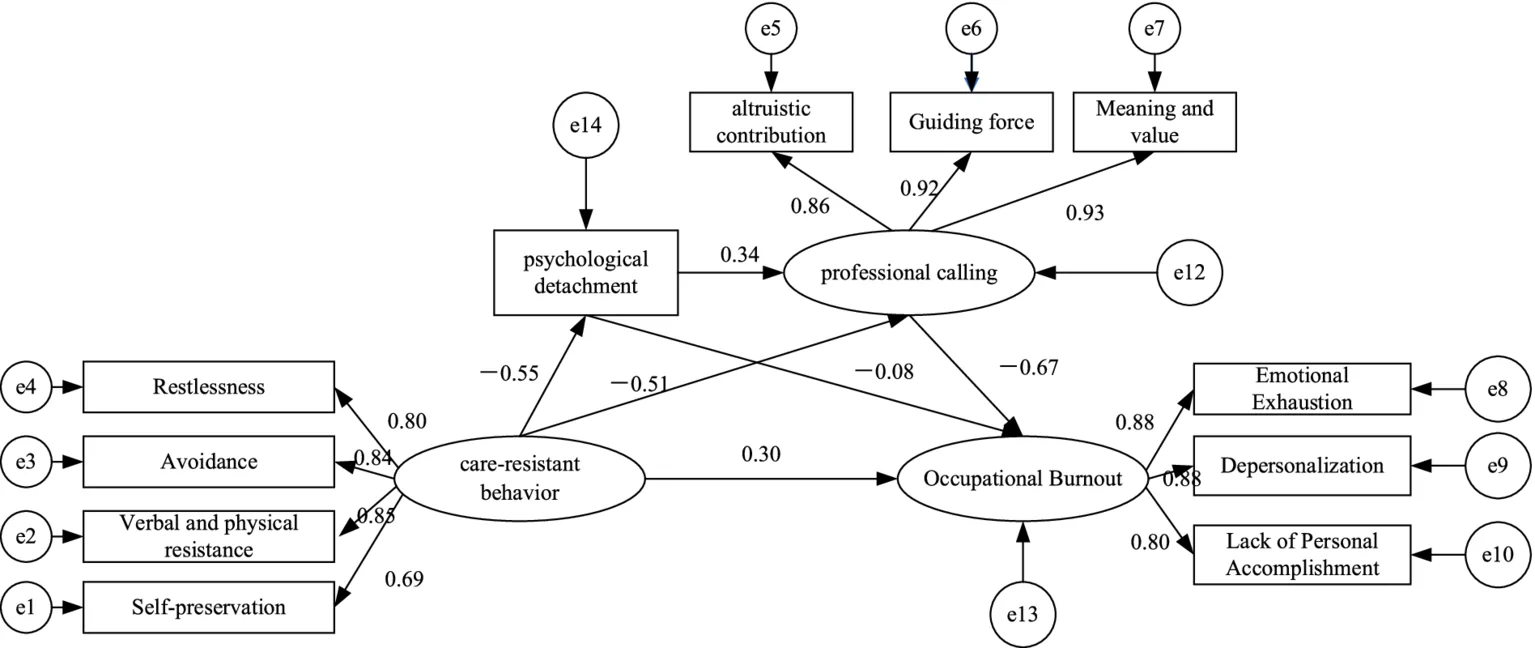
The hypothesized serial mediation model involving psychological detachment and professional calling.

The serial mediation analysis showed that the direct association and total indirect association between care-resistant behavior and nurses’ occupational burnout were statistically significant. The direct effect was 0.303, and the total indirect effect was 0.510 (0.044 + 0.340 + 0.126). Accordingly, the total effect was 0.813 (0.303 + 0.510). The direct effect and total indirect effect accounted for 37.3% and 62.7% of the total effect, respectively. The path results for each effect are presented in Table 1.

Care-resistant behavior → occupational burnout: Care-resistant behavior was directly and positively associated with occupational burnout. The effect size was 0.303, accounting for 37.3% of the total effect, indicating a positive effect of care-resistant behavior on occupational burnout.

Care-resistant behavior → psychological detachment → occupational burnout: With psychological detachment as a mediating variable, this specific mediation path did not reach a significant level, indicating that the independent mediating role of psychological detachment between the two was not established. The standardized indirect effect was 0.044 (Boot SE = 0.024, 95% bootstrap CI: −0.001–0.090), accounting for 5.4% of the total effect. Because the confidence interval included zero, this specific indirect effect was not statistically significant.

Care-resistant behavior → professional calling → occupational burnout: Professional calling significantly mediated the association between care-resistant behavior and occupational burnout. The specific indirect effect was 0.340, accounting for 41.8% of the total effect and 66.7% of the total indirect effect. The positive indirect effect indicated that greater exposure to care-resistant behavior was associated with greater occupational burnout through reduced professional calling.

Care-resistant behavior → psychological detachment → professional calling → occupational burnout: Psychological detachment and professional calling formed a significant serial indirect pathway between care-resistant behavior and occupational burnout. The serial indirect effect was 0.126. It accounted for 15.5% of the total effect and 24.7% of the total indirect effect. Greater exposure to care-resistant behavior was associated with lower psychological detachment, which was subsequently associated with lower professional calling and greater occupational burnout.

## 4 Discussion

This study explored the association between care-resistant behavior in dementia patients and nurses’ occupational burnout levels, while also examining the mediating roles of psychological detachment and professional calling. The main findings indicated that care-resistant behavior was directly associated with nurses’ occupational burnout. Additionally, it was indirectly associated with burnout via professional calling, as well as via a chain mediating pathway involving both psychological detachment and professional calling. However, the specific indirect effect transmitted solely through psychological detachment did not reach statistical significance. These results support an association consistent with care-resistant behavior as a typical social job demand and highlight the potential relevance of nurses’ internal psychological processes. Because all variables were measured concurrently, these findings do not demonstrate a causal mechanism.

The descriptive survey results showed that, in this study, the score for care-resistant behavior in dementia patients was 43.89 ± 21.75, which was higher than that reported in the study by Xiao et al. (2024). This finding suggests that care-resistant behavior is a relatively common and frequent phenomenon in the daily care of dementia patients, and nurses face a high risk of exposure to stressors in their daily work (Dall’Ora et al., 2020). Meanwhile, the occupational burnout score of nurses in this study was 63.00 ± 27.79, which was higher than that reported in the study by Luo (2025) on nurses’ occupational burnout. This study further suggests an inherent association between the high prevalence of care-resistant behavior in dementia patients and nurses’ occupational burnout levels. When patients frequently exhibit resistant, aggressive, or uncooperative behaviors, nurses not only need to invest more physical effort and time to complete basic nursing tasks, but also must continuously respond to sudden conflict situations. This dual burden of “emotional labor” and “physical risk,” accumulated over time, readily depletes nurses’ psychological resources, thereby elevating their levels of occupational burnout (Dall’Ora et al., 2020).

### 4.1 Direct association between care-resistant behavior in dementia patients and nurses’ occupational burnout levels

This study found that care-resistant behavior in dementia patients was directly and positively associated with nurses’ occupational burnout, with an effect size of 0.303, accounting for 37.3% of the total effect. Unlike patients in general wards, the resistant behaviors of dementia patients (such as refusing to eat, take medication, aggression, verbal abuse, wandering, etc.) are not driven by subjective malice, but are direct manifestations of cognitive impairment caused by their disease (Paggetti et al., 2025). However, this “non-subjective” nature does not alleviate the actual psychological impact on nurses; instead, due to its unpredictability and difficulty in communication, it leads to sustained depletion of nurses’ psychological resources (Holst and Skär, 2017). As a direct stressor, care-resistant behavior, if not buffered by sufficient work resources, can continuously trigger emotional exhaustion and depersonalization in nurses, thereby leading to occupational burnout.

According to the Job Demands-Resources (JD-R) theory, care-resistant behavior (CRB) in dementia patients can be defined as a typical and persistent job demand that continuously consumes nurses’ psychological and emotional energy (personal job resources), serving as a key stressor driving occupational burnout. On one hand, nurses need to invest additional time and effort to calm patients’ emotions and adjust care plans (Li et al., 2021); on the other hand, patients’ resistant behaviors may trigger feelings of frustration, anxiety, and even the accumulation of negative emotions in nurses (Nybakken et al., 2018). Prolonged exposure to such conditions gradually depletes nurses’ emotional regulation abilities, leading to manifestations of occupational burnout such as emotional exhaustion, depersonalization, and reduced personal accomplishment. This finding is consistent with the conclusions of Zhao et al. (2025) in their study of psychiatric nurses, which identified patients’ abnormal behaviors as one of the core risk factors for nurses’ occupational burnout. This study further focuses specifically on the “dementia patient” population, demonstrating a positive association between care-resistant behavior and nurses’ occupational burnout. From a practical perspective, these findings highlight several areas that may warrant consideration when developing future supportive interventions for nurses caring for patients with dementia. Potential approaches may include systematic specialized training in the management of care-resistant behavior, optimization of staffing and team support, and provision of psychological support and stress-management resources. However, the present observational findings do not demonstrate that these strategies would reduce occupational burnout, and their effectiveness should therefore be evaluated in appropriately designed intervention studies.

### 4.2 The mediating role of psychological detachment

This study found that the specific indirect association through psychological detachment in the relationship between care-resistant behavior in dementia patients and nurses’ occupational burnout was not significant, with a 95% confidence interval of −0.001 to 0.090. This result differs from recent nursing studies in which psychological detachment significantly mediated the associations of perceived social support or teamwork with burnout (Li et al., 2025; Cho et al., 2025) as well as evidence that psychological detachment mediated the relationship of workload with fatigue and sleep problems (Cho et al., 2024). A key distinction is that those studies examined organizational resources or relatively proximal recovery outcomes, whereas the present study examined an emotionally salient patient-related demand and the more distal outcome of occupational burnout.

The non-significant specific indirect effect should therefore not be interpreted as evidence that psychological detachment is irrelevant. In the serial model, the association between psychological detachment and burnout was estimated after professional calling had been included. Psychological detachment was moderately correlated with professional calling (*r* = 0.600), whereas professional calling showed a particularly strong negative correlation with burnout (*r* = −0.862). Accordingly, a substantial portion of the association involving psychological detachment may have been represented through professional calling, leaving only a small residual detachment-to-burnout pathway; this interpretation is consistent with the significant serial indirect effect and the non-significant detachment-only effect.

Psychological detachment is also a relatively proximal and fluctuating recovery experience, whereas professional calling and burnout reflect more enduring evaluations of work meaning and occupational strain. A single cross-sectional measurement may therefore be less sensitive to the short-term restorative effects of detachment. Moreover, the confidence interval for the detachment-only effect narrowly crossed zero (*β* = 0.044, 95% CI: −0.001 to 0.090), suggesting a weak or imprecisely estimated effect rather than definitive evidence of no association.

### 4.3 The mediating role of professional calling

The results of this study showed a statistically significant indirect association through professional calling in the relationship between care-resistant behavior in dementia patients and nurses’ occupational burnout, with a mediation effect value of 0.340, accounting for 41.8% of total effect. This finding is consistent with the results of Liu et al. (2025). Professional calling serves as a core driving force for nurses’ career development and a powerful source of intrinsic motivation. According to the JD-R theory, nurses with a high sense of professional calling (a job resource) can reinterpret the external pressure of care-resistant behavior (a high job demand) as a meaningful challenge rather than a burden, thereby enhancing emotional resilience through cognitive restructuring, strengthening intrinsic motivation and emotional stability, and achieving resource gain while alleviating occupational burnout (resource depletion). Research has shown that nurses with a high sense of professional calling perceive their work as meaningful, which reduces burnout and maintains high levels of motivation. Nurses with a strong sense of calling can maintain psychological balance even under pressure (Zhang et al., 2024). The relatively strong association between professional calling and occupational burnout (*r* = −0.862) warrants careful interpretation. Although these constructs are theoretically related, they represent distinct psychological dimensions: professional calling reflects nurses’ perceived meaning, purpose, and identification with their profession, whereas burnout reflects prolonged occupational strain characterized by emotional exhaustion, depersonalization, and reduced personal accomplishment. Nevertheless, because both constructs were assessed through self-report measures at the same time point, some overlap in item content or shared response tendencies may have contributed to the magnitude of their association. These findings indicate that professional calling may represent an important psychological resource associated with occupational burnout among nurses caring for patients with dementia. Accordingly, professional calling may be considered a potential target for future intervention development aimed at supporting nurses’ occupational well-being. Nevertheless, the present cross-sectional findings cannot establish that enhancing professional calling would lead to reductions in burnout. Whether interventions designed to strengthen professional calling can improve nurses’ occupational well-being and reduce burnout therefore requires direct evaluation in longitudinal or experimental studies.

### 4.4 The serial indirect association involving psychological detachment and professional calling

This study found a statistically significant serial indirect association involving psychological detachment and professional calling in the relationship between care-resistant behavior in dementia patients and nurses’ occupational burnout. The standardized serial indirect estimate was 0.126, accounting for 15.5% of the total association. The pathway was “care-resistant behavior → psychological detachment → professional calling → occupational burnout.” Care-resistant behavior in dementia patients is not merely simple non-cooperation, but is accompanied by multiple issues such as cognitive impairment, emotional dysregulation, and behavioral disturbances, characterized by persistence, suddenness, and unpredictability (Dero et al., 2023). This places nurses in a prolonged state of “high vigilance and high emotional involvement,” making it difficult for them to achieve effective psychological detachment (Cho et al., 2025). Chronic lack of psychological detachment leads to sustained depletion of nurses’ psychological resources, which indirectly affects their perception of professional value, resulting in a weakened sense of professional calling (Deng and Chen, 2019). Ultimately, under the dual effects of insufficient psychological resources and diminished professional motivation, nurses’ risk of occupational burnout significantly increases (Yuan et al., 2024). This process aligns with the JD-R theory: care-resistant behavior, as a job demand, continuously occupies psychological resources, leading to a spiral loss of resources. If this state extends beyond work hours and individuals cannot achieve effective psychological detachment, they remain in a state of latent work-related tension, unable to recover and replenish resources from work stress (Cho et al., 2024), which subsequently affects resource reserves (professional calling) and ultimately leads to the development of occupational burnout (Cho et al., 2024).

This pattern helps explain why psychological detachment was not significant as an isolated mediator but became significant in the serial pathway: its relevance emerged when the downstream association with professional calling was modeled. In other words, psychological detachment may be associated with lower burnout less through an immediate stand-alone route than by preserving the personal resources needed to sustain professional meaning, value, and commitment. Consistent with existing empirical evidence, psychological detachment is closely linked to reduced nurse burnout (Li et al., 2025; Cho et al., 2025), and professional calling can protect nurses against burnout by facilitating positive personal resources such as occupational resilience (Xie et al., 2024). Collectively, the present findings integrate these independent research conclusions, demonstrating that work recovery and professional meaning function sequentially rather than independently.

Notably, the serial indirect association observed in this study is consistent with the theoretically hypothesized sequence, in which greater care-resistant behavior corresponds to lower psychological detachment, lower psychological detachment correlates with weaker professional calling, and weaker professional calling further predicts higher occupational burnout. Within the JD-R framework, this pattern reflects a typical resource depletion process, wherein persistent occupational job demands impair individuals’ post-work recovery experiences and erode personal resources related to professional meaning. However, given the cross-sectional design with all variables measured concurrently, the identified statistical serial pathway cannot confirm that psychological detachment temporally precedes professional calling, nor can it verify that changes in these psychological constructs causally trigger subsequent alterations in occupational burnout.

Several alternative explanations are still plausible. For instance, nurses suffering from severe occupational burnout may simultaneously report diminished professional calling and impaired psychological detachment from work, and reciprocal and bidirectional relationships among these psychological variables are highly probable. Accordingly, the proposed serial pathway should only be regarded as a theory-consistent description of observed cross-sectional associations, rather than definitive evidence of a fixed temporal causal sequence. Longitudinal studies with repeated measurements, prospective cohort designs, or cross-lagged panel analyses are urgently required to verify the temporal precedence of variables and compare multiple theoretically reasonable directional relationships.

From an intervention perspective, the current findings indicate that psychological detachment and professional calling are promising targets for future burnout prevention research. Subsequent studies could specifically examine whether interventions targeting work recovery improvement and occupational meaning cultivation can optimize nurses’ psychological resources and alleviate occupational burnout. Before developing definitive clinical strategies and managerial recommendations, further validation of such intervention effects via longitudinal tracking or experimental designs is essential.

## 5 Limitations

*Limitations of the study*: First, this study employed a cross-sectional design, with all variables measured at a single time point; therefore, causal relationships and temporal precedence among care-resistant behavior, psychological detachment, professional calling, and occupational burnout cannot be established. The serial path model should consequently be interpreted as a theory-informed decomposition of contemporaneous associations rather than as evidence of a causal chain. Although the proposed ordering (care-resistant behavior → psychological detachment → professional calling → occupational burnout) was prespecified on the basis of the JD-R framework, alternative, reverse, reciprocal, and confounded relationships cannot be excluded. Accordingly, the hypothesized ordering should be viewed as theory-consistent rather than temporally established. Future prospective studies with repeated measurements, particularly cross-lagged panel or other longitudinal mediation designs, are needed to evaluate temporal precedence, compare theoretically plausible directional models, and clarify the dynamic relationships among these variables. Second, all variables were measured using self-report questionnaires. Although validated instruments, anonymous data collection, and the Harman single-factor test were used, recall bias, social desirability bias, and common method variance could not be completely ruled out. In particular, the strong correlation between professional calling and occupational burnout may partly reflect the conceptual proximity between these constructs or shared measurement characteristics. Future studies should consider incorporating multi-source assessments, temporal separation, or objective indicators to further reduce potential common method bias. Third, convenience sampling from five tertiary hospitals in a single province of China limits the representativeness and external validity of the sample. Moreover, the eligibility requirement of experiencing care-resistant behavior at least twice weekly may have introduced selection bias by preferentially including nurses with higher exposure levels. This exposure-enriched sampling strategy may have restricted the range of care-resistant behavior exposure and may limit the generalizability of the findings to nurses with lower exposure frequencies or different healthcare settings. Additionally, although several demographic and occupational characteristics showed significant associations with occupational burnout in preliminary analyses, these variables were not incorporated as covariates into the SEM model. Therefore, residual confounding caused by unmeasured demographic, occupational, or organizational factors cannot be completely excluded. Future studies should adopt theory-based covariate adjustment strategies, particularly in longitudinal designs, to further validate the observed associations. Consequently, the observed level of care-resistant behavior and the estimated associations should not be generalized to all nurses caring for people with dementia or to nurses working in primary care, community, and long-term-care settings. Consequently, the findings cannot be generalized to primary healthcare institutions, community nursing centers, or long-term care facilities. The manifestations of resistant behaviors in dementia patients, the sources of work stress for nurses, and the organizational support environment may vary across different healthcare settings. Future multicenter studies with stratified sampling are needed to validate the stability of the model. Additionally, the dynamic changes in psychological detachment and professional calling were not captured. Psychological detachment, as a recovery experience, may exhibit fluctuations in daily work, and professional calling may also evolve over the course of one’s career. This study was based on a single measurement and could not reveal the dynamic evolution of these variables over time or their cumulative effects on occupational burnout. Future research could employ experience sampling methods or diary studies to capture short-term fluctuations in variables at the daily or weekly level, thereby providing a more refined depiction of the chain mediation process.

## 6 Conclusion

This study included 421 nurses to examine the direct and serial indirect associations among care-resistant behavior, psychological detachment, professional calling, and occupational burnout. Given the cross-sectional nature of the data, these findings should be interpreted as theory-consistent associations rather than evidence of temporal or causal mechanisms.

From a practical perspective, the findings identify care-resistant behavior management, psychological recovery from work, and professional calling as potentially relevant domains for future burnout-prevention research. Nursing managers may consider these domains when developing and evaluating supportive programs, for example through specialized training in the management of care-resistant behavior, appropriate work and shift arrangements, psychological support resources, and activities aimed at reinforcing occupational meaning. However, the present study does not establish that modifying these factors would prevent or alleviate occupational burnout. The effectiveness of such approaches, as well as the hypothesized roles of psychological detachment and professional calling as intervention targets, should be tested in future longitudinal and intervention studies.

## Data Availability

The original contributions presented in the study are included in the article/Supplementary material, further inquiries can be directed to the corresponding author.
