# Supplementary material for: The association between care-resistant behavior in dementia patients and nurses’ occupational burnout: a cross-sectional serial mediation analysis of psychological detachment and professional calling
Source: Front Psychol. 2026 Sep 28;17:1860688. doi: 10.3389/fpsyg.2026.1860688 (PMC13655042; doi:10.3389/fpsyg.2026.1860688)
Supplement: Supplementary file 1 [file Table_1.DOCX]

Supplementary Table S1. Skewness and kurtosis values of the continuous variables

| Variable | Mean ± SD | Skewness | Kurtosis |
| --- | --- | --- | --- |
| Care-resistant behavior | 43.89 ± 21.75 | 0.674 | 2.096 |
| Psychological detachment | 9.61 ± 3.72 | 0.424 | -1.316 |
| Professional calling | 31.03 ± 10.96 | -0.419 | -1.359 |
| Occupational burnout | 63.00 ± 27.79 | 0.715 | -0.404 |
